# Assessment of Autonomic Nervous System Activity Using Spectral Analysis of Heart Rate Variability After Continuous Positive Airway Pressure (CPAP) Therapy in Patients With Sleep Apnea

**DOI:** 10.7759/cureus.51735

**Published:** 2024-01-06

**Authors:** Taisa Vasilkova, Valerie F Fiore, Alicia Clum, Angel Wong, Nawshin Kabir, Elizabeth Costello, Maxim Crasta

**Affiliations:** 1 Medicine, Lake Erie College of Osteopathic Medicine, Elmira, USA; 2 General Surgery, Lake Erie College of Osteopathic Medicine, Elmira, USA; 3 Physiology, Lake Erie College of Osteopathic Medicine, Elmira, USA

**Keywords:** cpap, sdnn, ans, sleep, obstructive sleep apnea, heart rate variability

## Abstract

Heart rate variability (HRV) measurements have emerged as a valuable tool for understanding the functioning of the autonomic nervous system (ANS) and assessing the health outcomes of obstructive sleep apnea (OSA) in patients. Sleep and the ANS exert a mutual influence on each other. Sleep promotes relaxation and recovery of the ANS. Conversely, ANS activity plays a role in regulating the onset and maintenance of sleep. The impact of continuous positive airway pressure (CPAP) therapy on patient recovery levels was investigated by assessing the restoration of ANS activity using HRV indicators. The study included patients with OSA who had been on CPAP for at least eight weeks. The patients were divided into two groups, namely the experimental group (CPAP-compliant) and the control group (CPAP-non-compliant). The study included a total of 38 patients, with 20 in the CPAP-compliant group and 18 in the CPAP-non-compliant group. The HRV analysis included time- and frequency-domain measures. Data was collected in various resting conditions, including lying down, standing, regular breathing, and under physiological stress induced by deep breathing and the Valsalva maneuver.

After CPAP treatment, there was an increase in the average values for SDNN for deep breathing and Valsalva maneuvers. The mean changes in SDNN for CPAP-non-compliant versus CPAP-compliant groups for normal breathing increased from 32.50±5.33 to 42.40±8.03, while the values for Valsalva increased from 20.16±2.47 to 25.45±3.03. Despite the observed variations in SDNN, there was no significant change in the average change in heart rate (∆ HR), except during the Valsalva maneuver. Post-CPAP values for the Valsalva ratio were significantly decreased in deep breathing. The E:I ratio for the CPAP-compliant group during normal breathing was 1.08±.16 compared to 1.55±.09; t (36) =-11.15, p <0.001 in the CPAP-non-compliant group. During deep breathing, the ratio was 1.36±.15 versus 1.59±.24; t (36) =-3.578, p <0.001. The high frequency (HF)nu mean values for deep breathing were 34.06±5.546 compared to 35.00±6.358; t (36) = -.485, p=.630. For the Valsalva maneuver, the values were 29.94±4.721 versus 26.95±6.621; t (36) =1.589, p=.060. The HF/low frequency (LF) ratio was found to be significant only in supine, standing, and normal breathing.

The utilization of CPAP therapy was found to be effective in achieving and sustaining autonomic balance during tasks like standing and engaging in regular breathing patterns. During activities that involve intense physical effort, like the Valsalva maneuver, the HRV metrics did not indicate any significant balance between sympathetic and parasympathetic activity. However, using CPAP therapy for a prolonged period can be beneficial in consistently improving the sympathovagal balance in these patients.

## Introduction

Untreated or undiagnosed obstructive sleep apnea (OSA) can result in an abnormal physiological state with significant health implications. This includes an elevated risk of developing cardiovascular disease, stroke, metabolic disorders, excessive daytime sleepiness, errors in the workplace, and an increased likelihood of being involved in traffic accidents [1,2]. According to studies investigating continuous positive airway pressure (CPAP) in daytime performance therapy, CPAP significantly improves both subjective and objective sleepiness, cognitive function, psychological well-being, and functional status. It is worth noting that CPAP is particularly advantageous for individuals with OSA because it aids in the restoration of physiological processes that facilitate the recovery of the autonomic nervous system (ANS) [3]. Various measures of ANS activity, including heart rate variability (HRV), have been used to examine the health implications related to the correlation between ANS activity and CPAP therapy in patients with OSA. Incomplete recovery of ANS activity has been demonstrated to have a detrimental impact on OSA patients' overall health after CPAP therapy. Research on this phenomenon aims to better understand its impact. Heart rate variability measurements provide valuable insight into the functioning of the ANS and the health outcomes of OSA patients in this context [4-6]. Recently, several studies have used HRV analysis to investigate the underlying connection between ANS activity and OSA [7]. Due to its non-invasive nature, versatility, and sensitivity to both physiological and environmental stimuli, HRV is often used to indicate changes in the ANS.

The relationship between sleep and the ANS is reciprocal, meaning that they have a mutual influence on each other [8]. It has been shown that reduced HRV leads to higher morbidity and mortality due to the worsening of various conditions such as hypertension, heart disease, and increased susceptibility to stress [9]. Conversely, increases in HRV can lead to lowered stress levels, better sleep, and enhanced self-regulation capacities, as well as improved executive functions such as processing emotions and paying attention [10]. The use of CPAP helps patients with sleep-disordered breathing because it restores the physiological processes that promote the central nervous system's recovery. Previously, CPAP therapy has been shown to significantly improve subjective and objective sleepiness, cognitive function, psychological well-being, and functional status among patients [3]. Consequently, patients who undergo this therapeutic intervention notice improved neurocognitive abilities and overall wellness.

The ANS plays a critical role in HRV's relationship with physiological changes that can be measured and correlated to health indices. The efferent nerve impulses of the vagal and sympathetic systems establish this relationship [11]. The heart rate experiences periodic fluctuations when a person is at rest or has OSA, which occurs due to the dominance of either the sympathetic or parasympathetic nervous systems [12]. Many research studies have documented the variations in vagal outflow, which give rise to cyclic fluctuations of high frequency (HF) and disrupt the balance between sympathetic and parasympathetic activities, leading to worsening states in pathological situations. These fluctuations in heart rate can serve as an indication of an imbalance in the autonomic control system [13,14]. There have not been a significant number of studies that have focused on investigating the effects of CPAP therapy on the ANS. Therefore, the emphasis of the current study is placed on the physiological impact of this therapy.

This study aimed to determine the impact of CPAP therapy on individuals who have received treatment for at least eight weeks by employing the time domain and frequency domain measures of HRV and to determine whether CPAP significantly restored autonomic function in patients. We hypothesize that CPAP therapy helps restore autonomic balance in patients with sleep-disordered breathing by helping them regain physiological processes that facilitate ANS recovery.

An abstract of this study was previously presented at the Lake Erie College of Osteopathic Medicine (LECOM) Research Day on October 28, 2022.

## Materials and methods

Subject selection

This study was approved by the Institutional Review Board of the Lake Erie College of Osteopathic Medicine, Elmira, NY, USA (approval no. 28-10). Study participants were patients attending Arnot Ogden Medical Center's Sleep Disorder Center and Pulmonology Clinic in Elmira, NY, USA. Patients who had been diagnosed with OSA by their physician and had completed at least eight weeks of CPAP therapy were assigned to the experimental group using a cross-sectional design. Patients who were diagnosed with a sleep disorder or OSA but did not adhere to the recommended CPAP therapy during the follow-up visit were recruited for the CPAP non-compliant group. A total of 38 patients were included in the study after meeting the criteria given below. The CPAP-compliant group included 20 patients, and the CPAP-non-compliant group comprised 18 patients, which served as the comparison group. The number of participants was determined by utilizing the sample size calculation procedure employed by earlier researchers [15]. To ensure a homogeneous and valid sample, 38 individuals who visited a sleep center and met all the inclusion and exclusion criteria were selected. None of the patients were excluded once they were enrolled in the study.

To obtain accurate and dependable data as well as their corresponding parameters, the study recruited individuals who were diagnosed with OSA but did not have any underlying conditions that could lead to autonomic dysfunction. Exclusion criteria included patients who were pregnant or lactating, patients with physical or mental impairments, night-shift workers, patients with underlying diseases causing autonomic dysfunction and cardiac arrhythmia, evidence of chronic lung disease, patients taking any beta-blockers or antiarrhythmic drugs, and those who used a stimulating substance like alcohol, caffeine, nicotine, or an energy drink within two hours of data collection. Patients who smoke or use recreational drugs and have severe adverse or allergic reactions to contact with metal or plastic were also excluded. As soon as the healthcare provider met Medicare guidelines for CPAP therapy, the patient was referred to our research team. According to Medicare guidelines, CPAP compliance is to be determined by a face-to-face evaluation with a caregiver. To be compliant, the patient must use the CPAP machine for at least four hours each night, i.e., 70% of the total night. Everyone else was considered non-compliant. The study team members focused on individuals who underwent a new face-to-face evaluation with a healthcare professional, followed the 'new patients' steps, and returned for follow-up after eight weeks of utilization. Only those who returned to the hospital during this period were included in the study. Participants were excluded if they had lung diseases, cardiovascular diseases, or any other criteria of exclusion to ensure that the data was not skewed across different severity levels of OSA. Conditions such as these were used as exclusion criteria, preventing individuals from being included. A quiet room with a regulated temperature was provided to create an optimal environment, while specific instructions were given to the patients, like speaking to minimize the external influences on the ANS.

Intervention protocol

Spectral analysis of the data was performed using HRV software developed by IntelleWave Inc., Largo, FL, USA. The IntelleWave HRV system measures HRV quantitatively with a fully automated system. Physiological data can also be obtained using IntelleWave. Using this software, HRV activity can be monitored through real-time quantitative measurements of the spectral function components of HRV. By enabling both graphical and digital visualization of HRV analysis, this software provides a quantitative evaluation of HRV analysis. This device uses an electrocardiographic signal recorded from a chest strap. The strap is securely placed on the participant's chest at the xiphoid process. To collect the data, a transmitter belt (Polar® T31 coded™ transmitter, Polar Electro Oy, Kempele, Finland) was used. The recorded data was then wirelessly transmitted via Bluetooth to a computer for processing and storage. During the HRV measurement process, participants were initially instructed to relax while lying down for 10 minutes. Subsequently, the following six minutes were used for data collection.

The participants were instructed to carry out a series of tasks following the fastening of the chest trap with the software in operation. These tasks included lying on their backs with their eyes closed but remaining awake, standing upright, maintaining normal breathing, taking deep breaths, and finally, completing the Valsalva maneuver while gripping a caliper. All tests were noninvasive and typically took 10 minutes to complete. The ANS activities were measured accurately and reliably following these standardized procedures. Guidelines are in place to discourage engaging in conversation or any other activities that may introduce external disturbances. A self-administered Epworth sleepiness scale and Beck’s depression scale were used to collect data about daytime somnolence and psychological well-being, respectively, and the data obtained was not included in this article.

To assess the variability of HRV in the time domain, various parameters were recorded and entered into a data sheet. These parameters included the standard deviation of NN intervals (SDNN), the change in heart rate (∆ HR), the Valsalva ratio, and the expiration (E):inspiration (I) ratio. These measures were recorded while the patients were engaged in the designated tasks. The Valsalva ratio was computed by dividing the longest R-R interval observed after performing the Valsalva maneuver with the shortest R-R interval recorded during the hand grip maneuver. The E:I ratio was computed by taking the longest RR interval during expiration and the shortest RR interval during inspiration, and then calculating the ratio between them.

The measurements in the frequency domain were taken in the range of 0.01 to 0.40 Hz. The power within the low-frequency (LF) range was defined as 0.04 to 0.15 Hz, while the power within the high-frequency range was defined as 0.15 to 0.40 Hz. To accurately analyze the LF/HF balance while minimizing the impact of changes in total power, the LF and HF indices were normalized. The LF normalized units (LFnu) were calculated by multiplying the LF power by 100 and dividing it by the sum of the HF and LF powers. Similarly, the HF normalized units (HFnu) were obtained by multiplying the HF power by 100 and dividing it by the sum of the HF and LF powers. To determine the LF/HF ratio, an Excel spreadsheet (Microsoft Corp., Redmond, WA, USA) was used.

Statistics

The statistical software SPSS Statistics version 29.0 (IBM Corp., Armonk, NY, USA) was utilized for data analysis in this study. Continuous variables were presented as the mean ± standard error of the mean (SEM). A significance level of p <0.05 was chosen to determine statistical significance. To select the appropriate statistical test, the normality of the data was evaluated. If the data exhibited a normal distribution, an independent sample t-test was employed. On the other hand, if the data did not follow a normal distribution, the Mann-Whitney U test was utilized. The assumption of homogeneity of variance was assessed using Levene's test of equality of variances. This specific test was produced within the SPSS Statistics software during the execution of the independent t-test procedure.

## Results

In Table 1, a comparison is made between the time-domain HRV measures obtained from the independent sample tests for the controls and CPAP-treated groups. Based on the results of the ANS test, mean values observed in patients who received CPAP therapy exhibited significant variation based on t-values or confidence intervals. Patients' HRV metrics both varied in frequency and time domain, according to the study findings.

**Table 1:** Time domain measures of HRV in CPAP-non-compliant (control ) and CPAP-compliant patients The HRV metrics of the CPAP-compliant and CPAP-non-compliant groups' mean ± SEM values are summarized. Mean values with p <0.05 are statistically significant. HRV: Heart rate variability; ANS: Autonomic nervous system; CPAP: Continuous positive airway pressure; SEM: Standard error of the mean; SDNN: Standard deviation of normal NN interval; ∆ HR: Heart rate variations minimum and maximum; DBreathing: Deep breathing; NBreathing: Normal breathing; Val: Valsalva; EI: Expiration to inspiration ratio

Independent samples test							
ANS test	CPAP non-compliant mean ± SEM	CPAP compliant mean ± SEM	t-value	Significance		95% confidence interval of the difference	
				One-sided p	Two-sided p	Lower	Upper
Supine_SDNN	29.94±6.70	33.45±5.22	-1.807	0.040	0.079	-7.44	0.42
Standing_SDNN	32.44±7.03	32.50±5.01	-0.028	0.489	0.978	-4.04	3.93
NBreathing_SDNN	32.50± 5.33	42.40± 8.03	-4.420	<0.001	<0.001	-14.44	-5.35
DBreathing_SDNN	36.83± 4.90	37.05± 4.72	-0.139	0.445	0.891	-3.38	2.95
Valsalva_SDNN	20.16± 2.47	25.45± 3.03	-5.837	<0.001	<0.001	-7.11	-3.44
Supine_∆ HR	9.28±1.81	10.25±1.77	-1.672	0.052	0.103	-2.15	0.20
Standing_∆ HR	10.33±1.75	11.20± 2.40	-1.261	0.108	0.216	-2.26	0.52
NBreathing_∆ HR	12.28±2.97	12.65±4.80	-0.283	0.389	0.778	-3.03	2.29
DBreathing_∆ HR	10.83±2.43	11.35±1.35	-0.821	0.209	0.417	-1.79	0.75
Valsalva_∆ HR	11.39±1.65	10.05±2.09	2.175	0.018	0.036	0.09	2.58
Supine_Val Ratio	1.20±0.14	1.18±0.17	0.476	0.318	637	-0.083	0.42
Standing_Val Ratio	1.03±0.15	1.05±0.18	0.481	0.339	0.677	-0.13	3.93
NBreathing_Val Ratio	1.081±0.21	1.13±0.21	-0.419	0.251	0.503	-0.18	2.95
DBreathing_Val Ratio	1.60±0.07	1.47±0.12	-0.424	<0.001	<0.001	0.07	-5.35
Valsalva_ Val ratio	1.49±0.13	1.69±0.06	4.49	<0.001	<0.001	0.07	-3.44
Supine_EI ratio	1.11±.20	1.18±.08	-1.463	0.076	0.152	-0.17	0.02
Standing EI ratio	1.18±.16	1.20±.12	-0.608	0.274	0.547	-0.11	0.06
NBreathing_EI ratio	1.08±.16	1.15±.09	-11.148	0.164	<0.001	-0.54	-0.37
DBreathing_EI ratio	1.36±.15	1.59±.24	-3.578	<0.001	0.001	-0.37	-0.10
Valsalva _EI ratio	1.21±.14	1.25±.18	-0.703	0.243	0.486	-0.14	0.06

**Figure 1:**
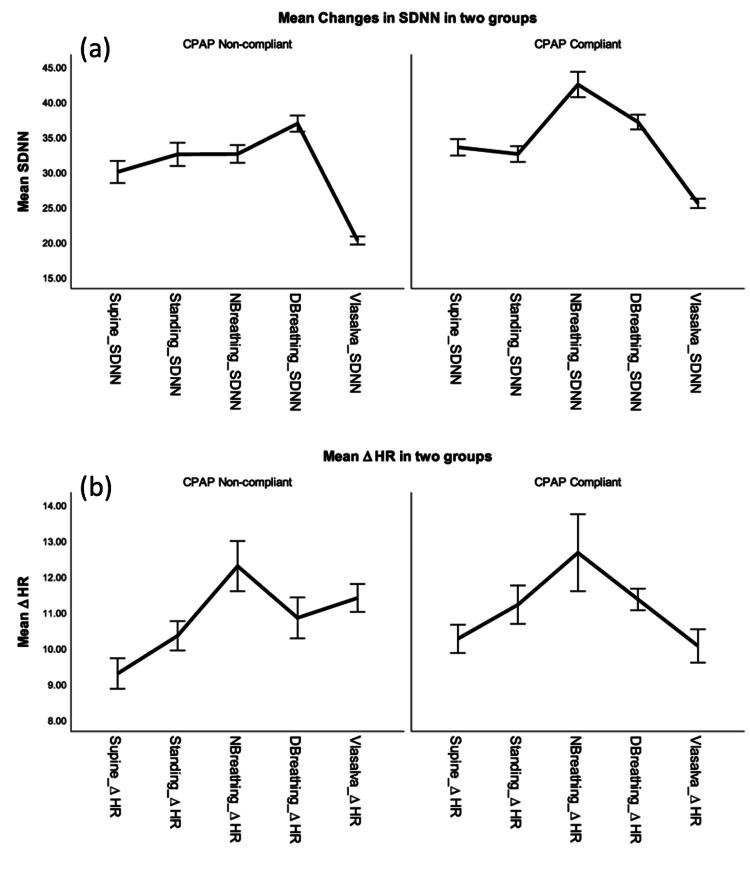
A comparison between the mean changes in SDNN and the ∆ HR in CPAP-noncompliant and CPAP-compliant groups. (a) The impact of different autonomic tests on the variations in minimum and maximum heart rates (∆ HR); (b) The corresponding standard deviation of the heart rate variability (SDNN). The normal breathing and Valsalva maneuvers revealed statistically significant differences in ∆ HR and SDNN, indicating a positive influence of CPAP therapy on HRV metrics. HRV: Heart rate variability; CPAP: Continuous positive airway pressure; NBreathing: Normal breathing; DBreathing: Deep breathing

The average values for normal breathing in the CPAP-non-compliant group versus the CPAP-compliant group showed an increase from 32.50±5.33 to 42.40±8.03, while Valsalva demonstrated an increase from 20.16±2.47 to 25.45±3.03, respectively. The changes were statistically significant at a p <0.001. Figure 1 illustrates the impact of CPAP treatment on the SDNN values for both normal breathing and Valsalva breathing techniques. The results indicate a notable average increase in SDNN values, accompanied by a negative 95% confidence interval. These findings suggest a statistically significant improvement in ANS function following CPAP treatment. The observed increase in SDNN values supports the notion that CPAP therapy positively affects HRV metrics. The results indicate that the use of CPAP therapy has a beneficial effect on the regulation of the ANS during normal breathing and when performing the Valsalva maneuver. The ∆ HR suggests that there was no substantial difference in the average heart rate. By using a two-sided p-value to compare the ∆ HR between the CPAP-compliant group and the CPAP-non-compliant group, it was observed that neither group had significant changes, except for the Valsalva group (p = 0.036), where this decreased in the CPAP-compliant group. The concurrent decrease in SDNN and reduced ∆ HR suggest (as shown in Figure 1) that ANS might not be efficiently regulating changes in the HRV in the Valsalva group.

**Figure 2:**
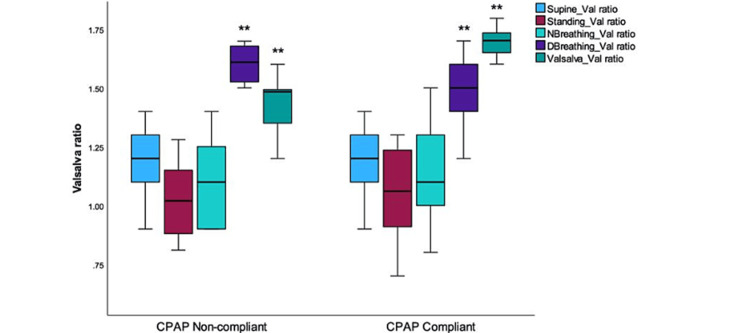
The Valsalva ratio values obtained from ANS testing were compared between CPAP-noncompliant and CPAP-compliant groups. This ratio serves as a time domain measurement of HRV. The graph presents the distribution of values and the range of Valsalva ratio outcomes obtained during the testing period. The ratio was determined by computing the lengths of the longest and shortest RR intervals determined during various autonomic tests. A significant difference was observed between the deep breathing and Valsalva groups of patients, specifically when analyzing CPAP groups (** indicates significance level p <0.001. ANS: Autonomic nervous system; CPAP: Continuous positive airway pressure; HRV: Heart rate variability; NBreathing: Normal breathing; DBreathing: Deep breathing; Va;: Valsalva

The ANS test results are presented in Figure 2, indicating that only two values showed a significant difference between the two groups. The ratio recorded during deep breathing was 32.50±5.34 for CPAP-non-compliant patients, whereas it was 42.40±8.04 for CPAP-compliant patients. This difference in ratios was found to be statistically significant (p <0.001). Similarly, the computed values during the Valsalva maneuver were also significant.

For CPAP-non-compliant patients, the ratio for the Valsalva group was 20.17±2.48. On the other hand, CPAP-compliant patients had a ratio of 25.45±3.03. These findings highlight the distinct increase in the computed values in the CPAP-compliant group (the negative t value indicates the effect of the intervention) during the Valsalva maneuver.

**Figure 3:**
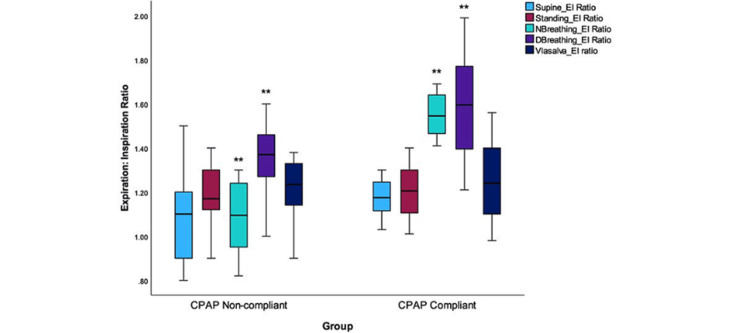
The E:I ratio in the CPAP-noncompliant and CPAP-compliant groups The graph represents the distribution of values of E:I ratios in the two CPAP groups. The values were computed while each subject executed maximal deep-breathing procedures while simultaneously recording HRV. The E:I ratio was determined by calculating the mean of the longest RR interval during maximal expiration and the mean of the shortest during maximal inspiration. A significant difference was observed between the normal and deep breathing groups (**indicates significance level p <0.001). E:I: Expiration to inspiration; CPAP: Continuous positive airway pressure; HRV: Heart rate variability; NBreathing: Normal breathing; DBreathing: Deep breathing

The E:I ratio was calculated using the average of inspiration and expiration expressed in terms of R-R intervals. A significant difference was observed in only breathing maneuvers, and this was found to be the only significant difference noted (Figure 3). This was 1.36±.15 versus 1.59±.24 during deep breathing; t (36) = -3.578, p <0.001. A significant effect of inspiration-mediated vagal dominance on HRV was observed in this group based on E:I change.

Frequency domain measures

The CPAP treatment and controls are compared in Table 2 using the frequency domain measures from the independent sample tests. 

**Table 2:** Summary of the frequency domain analysis results The CPAP-compliant and CPAP-non-compliant groups' mean ± SEM values are compared. Mean values with p <0.05 are statistically significant. The ratios indicate a calculated ratio of LF to HF. CPAP: Continuous positive airway pressure; SEM: Standard error of the mean; DBreathing: Deep breathing; NBreathing: Normal breathing; LFnu: Low frequency normalized units; HFnu: High frequency normalized units

Frequency domain analysis	CPAP non-compliant mean ±SEM		CPAP compliant mean ±SEM	t-value	Significance		95% confidence interval of the difference	
					One-sided p	Two-sided p	Lower	Upper
Supine_HFnu	35.56±3.633		49.50± .916	-8.635	<0.001	<0.001	-17.219	-10.669
Standing_HFnu	29.78± .474		51.75± .582	-0.484	<0.001	<0.001	-17.298	-18.645
NBreathing_HFnu	36.00±6.426		44.50±5.916	-4.246	<0.001	<0.001	-12.560	-4.439
DBreathing_HFnu	34.06±5.546		35.00± 6.358	-0.485	0.315	0.630	-4.890	3.001
Valsalva_HFnu	29.94±4.721		26.95±6.621	1.589	0.060	0.121	-0.828	6.817
Supine_LFnu	66.67±9.834		56.30±13.849	2.633	0.006	0.012	2.380	18.352
Standing_LFnu	78.78±5.786		57.15±15.652	5.526	<0.001	<0.001	13.690	29.564
Nbreathing_LFnu	73.22±9.059		63.85±8.641	3.263	0.001	0.002	3.546	15.197
DBreathing_LFnu	62.22±12.628		63.35±9.980	-0.307	0.380	0.761	-8.579	6.323
Valsalva_LFnu	96.39±14.880		91.45±14.659	1.030	0.155	0.310	-4.789	14.667
Supine_ratio	3.45±.384		2.74±.417	5.399	<0.001	<0.001	0.440	0.969
Standing_ratio	3.94±.255		3.40±.260	6.447	<0.001	<0.001	0.369	0.708
NBreathing_ratio	3.54±.450		3.17±.413	2.655	0.006	0.012	0.087	0.656
DBreathing_ratio	3.59±.191		3.59±.567	-0.008	0.497	0.994	-0.286	0.283
Valsalva_ratio	3.87±.309		3.80±.299	0.676	252	0.504	-0.133	0.266

**Figure 4:**
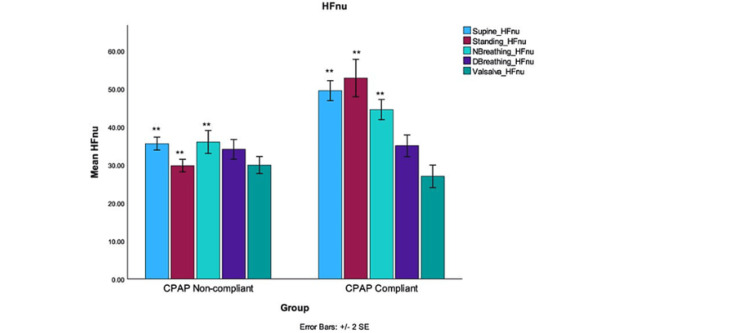
The calculated mean HFnu values were derived from the analysis of the HF spectral components of HRV compared between the two groups. The graph depicts a significant difference in the HFnu values among the supine, standing, and normal breathing groups (**indicates significance level p <0.001). The mean HFnu values in the CPAP group were found to be increased, suggesting a potential beneficial effect of CPAP therapy due to dominant parasympathetic modulation. CPAP: Continuous positive airway pressure; DBreathing: Deep breathing; NBreathing: Normal breathing; LFnu: Low frequency normalized units; HFnu: High frequency normalized units

The calculated HFnu values showed no significant improvement when patients were subjected to autonomic challenges such as deep breathing, or the Valsalva maneuver (Figure 4).

**Figure 5:**
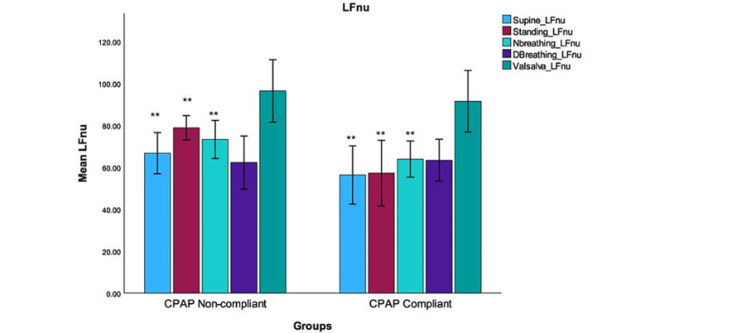
The LFnu are calculated values obtained through LF spectral analysis of HRV in both the CPAP-non-compliant and CPAP-compliant groups. The calculated LFnu values are an index of the modulation of the sympathetic branch of the ANS, which showed a significant decrease in the supine, standing, and during normal breathing maneuvers in CPAP-compliant groups (**indicates significance level p <0.001). CPAP: Continuous positive airway pressure; DBreathing: Deep breathing; NBreathing: Normal breathing; LFnu: Low frequency normalized units; HFnu: High frequency normalized units

The mean values for deep breathing for CPAP-non-compliant were 34.06±5.546 and the mean values for CPAP-non-compliant were 35.00± 6.358; t (36) = -0.485, p = 0.630. The mean values for the Valsalva maneuver for the CPAP-compliant group were 29.94±4.721 versus 26.95±6.62; t(36) = 1.589, and p = 0.060 for the non-compliant group. The LFnu, as shown in Figure 5, reflects the combined sympathetic and parasympathetic responses to autonomic testing and exhibited a significant decrease in the CPAP-compliant group, except for deep breathing and Valsalva maneuver tests (p = 0.38 and 0.15, respectively).

**Figure 6:**
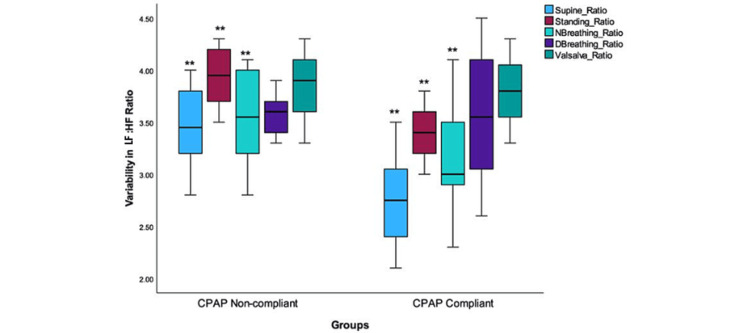
The variability in LF:HF Ratio in the CPAP-non-compliant and CPAP-compliant groups The LF:HF ratio reflects the sympathovagal balance. The boxplots exhibit significantly different median values in each of the groups: supine, standing, and normal breathing. The median values (**indicates significance level p <0.001) also exhibit a high degree of variability. A decreased LF:HF ratio in the CPAP groups indicates high parasympathetic activation, indicating a positive impact on the CPAP-compliant group during these tests. CPAP: Continuous positive airway pressure; DBreathing: Deep breathing; NBreathing: Normal breathing; LF: Low frequency; HF: High frequency

These findings suggest that the CPAP-compliant group experienced altered autonomic responses when subjected to deep breathing and Valsalva maneuver tests, as indicated by the non-significant changes in LFnu values. A comparison of ANS activity balance after CPAP therapy is illustrated in Figure 6.

Specifically, the LF:HF ratio was found to be significant while in a supine position, while standing, and during normal breathing (p <0.001). However, it is worth noting that no significant difference in the LF:HF ratio was observed during deep breathing or the Valsalva maneuver. The graphical representations in Figures 5 and 6 show a significant reduction in values among the CPAP-compliant group during various normal physiological activities. These activities included supine, standing, and normal breathing for patients in the CPAP-compliant group. This reduction was found to be statistically significant (p <0.001). The normalized units of the LFnu component, serving as a quantitative marker of sympathetic modulation, were also examined. The LF:HF ratio calculation indicated a decrease in ratio, indicating a notable reduction in sympathetic activity accompanied by increased parasympathetic activity. This significant decrease in sympathetic and an increase in parasympathetic activity was evident in all of the participants, regardless of these groups (p <0.001).

## Discussion

Previous research demonstrated a correlation between OSA and the interference of both the sympathetic and parasympathetic nervous systems, but it appears that this effect can be reversed through the administration of suitable treatment for OSA [16]. The measurement and analysis of HRV have proven to be valuable non-invasive techniques for assessing cardiac autonomic functions, which regulate heart rate by means of parasympathetic and sympathetic activity.

Based on the consequential increases in HFnu and LF:HF ratios among CPAP users, it is apparent that there is a great deal of parasympathetic tone dominating among CPAP users. The LF:HF ratio serves as a valuable tool for assessing the balance between the sympathetic and parasympathetic autonomic functions. This calculation involves dividing the LF component by the HF component, which is obtained from the readings provided by the software. The assessment of the impact of CPAP therapy on different physiological factors in individuals with OSA involved the analysis of several metrics. One such metric was the SDNN, which refers to the variability of time intervals between consecutive heartbeats. These findings indicate that the ANS influence remained strong and consistent over the entire observation period. Interestingly, following the commencement of CPAP therapy for eight weeks, most of the HRV time domain parameters did not show any significant alterations, except for deep breathing and the Valsalva maneuver. Significant differences were not detected while in the supine position, standing, or during regular breathing. There is a possibility that the utilization of deep breathing techniques could lead to the expansion of lung tissues, thereby causing the activation of vagus nerve endings and enhancing the parasympathetic nervous system. Consequently, this could potentially increase SDNN. The improved parasympathetic dominance suggests a transition towards a more balanced autonomic response. The restoration of the ANS and the variability of SDNN may depend on the length of the treatment and the seriousness of the apnea, as previously proposed [17,18].

Additionally, we explored other time-domain measures, including alterations in heart rate, the E:I ratio, and the Valsalva ratio. The E:I ratio is a measure of the time spent exhaling compared to the time spent inhaling during the deep breathing test. An increase in bursts in either the sympathetic or parasympathetic systems can lead to a significant increase in the E:I ratio. This indicates a shift towards parasympathetic dominance, as the parasympathetic system is responsible for controlling the exhalation phase of breathing. Similarly, an increase in bursts in either the sympathetic or parasympathetic system can significantly impact the Valsalva ratio, indicating changes in autonomic control of the cardiovascular system.

Our results indicated a rise in the prevalence of parasympathetic dominance and a reduction in sympathetic dominance. This is evident from the frequency domain measures, including Hfnu, LFnu, and the LF:HF ratio. Specifically, there was a notable decrease in the LF:HF ratio. These results indicate a shift towards a more parasympathetic state and a decrease in sympathetic activity among the CPAP-compliant patients. In previous work, the interpretation of normalized spectral HRV indices was also found to be similar to what this study describes [19,20]. Figures 2-4 show the impact of deep breathing and Valsalva maneuvers on HRV measures. The difference in the CPAP effect may be attributed to a decrease in sympathetic activity and the temporary dominance of parasympathetic activity during rest. This is indicated by time-domain measures. These findings suggest that sympathetic activity decreases while CPAP promotes vagal tone, which helps restore the balance of the ANS. However, it is significant to note that CPAP-compliant patients exhibit a lower LF:HF ratio, indicating predominant parasympathetic activity during the supine position, normal breathing, and standing.

The method used to quantify the outflows of the ANS is widely employed. However, it does raise certain methodological concerns, as indicated by a study by Heathers et al. [20]. The interpretation of LFnu and HFnu, as well as the ratio LF:HF, are influenced by external factors such as the time of day of recording and age factors. Despite these concerns, the method appears to demonstrate good levels of reproducibility. In fact, the reproducibility levels are comparable to those reported in previous research studies [21,22]. The frequency bands analyzed in this study align with the previous guidelines outlined by the American Heart Association, which provide recommendations for the measurement and interpretation of HRV [23].

The effects of certain interventions do impact the ANS functions, as reported in the literature. Numerous therapeutic approaches have been examined in the past to hinder or delay the advancement of cardiovascular autonomic dysfunction. Certain interventions, such as maintaining tight control over glycemic levels and utilizing antioxidants, have demonstrated some enhancement in autonomic function [24,25]. It has been demonstrated that an elevation in hypoxia and hypercapnia during sleep triggers an upsurge in sympathetic nerve activity. This physiological response is primarily mediated by chemoreflexes [26], which in turn may result in heightened renin-angiotensin activity [27]. It is plausible that deep inspiration could potentially stimulate the vagus nerve through the stretch receptors in the lungs. This indicates that the changes in heart rate observed might have potentiating effects on the parasympathetic nervous system. There is evidence to suggest that breath excursions may be exerting a potentiating effect on the parasympathetic nervous system [28].

In the short term, treating OSA-induced ANS dysfunction can have immediate positive effects on an individual's well-being. This might primarily be attributed to the restoration of adequate oxygenation during sleep, which facilitates the normal functioning of the ANS. There is evidence pointing to a dose-response relationship between the severity of OSA and the activation of the sympathetic nervous system [29]. Overall, by examining both the time domain and frequency domain analyses of HRV, we can gain a comprehensive understanding of the relationship between HRV and the parasympathetic effects that arise from CPAP therapy.

Limitations

The present study is subject to certain limitations. The individuals partaking in the study were not randomly chosen from the general population, and there was a higher representation of men compared to women. Our findings indicate that when assessing stress through short-term HRV measurement, it is essential to consider age and gender as significant factors. The effectiveness of CPAP treatment can vary depending on various factors, including the severity of the sleep disorder, stress, physical activity, emotional state, the overall health of the individual, and adherence to the treatment regimen. This can pose challenges in obtaining consistent and reliable results when using HRV as the sole measure of CPAP treatment effectiveness. Furthermore, the lack of standardized protocols for HRV measurement and the inability of HRV to capture the entirety of cardiovascular function further restrict its utility in studies on CPAP treatment.

## Conclusions

The evaluation of sympathetic and parasympathetic activity suggests that the utilization of CPAP therapy may have the potential to regulate the equilibrium and rhythm of the ANS. This regulation is demonstrated through the analysis of both the time and frequency domains of HRV. When individuals perceive an increase in well-being and experience an elevation in HRV, it indicates a positive impact of CPAP therapy on the regulation of the ANS. During regular activities such as lying down, standing, and normal breathing, there is a predominance of parasympathetic activity observed in the frequency domain parameters. However, tasks requiring significant physiological effort, such as the Valsalva maneuver, may not exhibit a restoration of balanced sympathovagal activity after CPAP therapy. These findings suggest that a more extended duration of CPAP therapy could potentially be advantageous, as it holds the potential to reinstate autonomic balance during strenuous activities.
